# Association between platelet-to-high-density lipoprotein cholesterol ratio and incident diabetes risk: a national representative cohort study based on CHARLS

**DOI:** 10.1186/s12902-026-02627-x

**Published:** 2026-10-12

**Authors:** Lizhu Chen, Chengpeng He, Xiaolu Chen

**Affiliations:** 1 Department of Cardiology, Cardiovascular Center, Beijing Friendship Hospital, Capital Medical University, Beijing, P.R. China; 2 Department of Pediatrics, Chengdu Second People’s Hospital, Chengdu, Sichuan Province P.R. China; 3 Department of Pediatrics, West China Second University Hospital of Sichuan University, Key Laboratory of Obstetric & Gynecologic and Pediatric Diseases and Birth, Defects of Ministry of Sichuan University, Chengdu, Sichuan province P.R. China

**Keywords:** Platelet-to-high-density lipoprotein cholesterol ratio, Diabetes, CHARLS

## Abstract

**Background:**

Diabetes mellitus has become a major global public health burden, particularly in China. The platelet-to-high-density lipoprotein cholesterol ratio (PHR) has emerged as a novel inflammatory and metabolic biomarker associated with cardiometabolic risk. However, prospective evidence regarding its association with incident diabetes remains limited. This study aimed to investigate the association between PHR and the risk of incident diabetes among Chinese middle-aged and older adults.

**Methods:**

This prospective cohort study used data from the China Health and Retirement Longitudinal Study (CHARLS) spanning from 2011 to 2020. A total of 5835 participants without diabetes at baseline (2011–2012) were included and followed up in 2013, 2015, 2018, and 2020. Participants were divided into four groups according to PHR quartiles. Kaplan-Meier survival analysis and Cox proportional hazards regression models were employed to evaluate the association between PHR and incident diabetes. Restricted cubic spline (RCS) regression was performed to explore potential linear and non-linear relationships. Subgroup and sensitivity analyses were conducted to assess the robustness of the findings.

**Results:**

During a median follow-up of 116 months, 669 participants developed incident diabetes. Kaplan–Meier analysis revealed a significantly higher cumulative incidence of diabetes in higher PHR quartiles (log-rank *p* < 0.001). After multivariable adjustment, Cox regression showed that participants in the highest PHR quartile (Q4) had a significantly increased risk of diabetes compared with those in the lowest quartile (Q1) (adjusted hazard ratio (HR) = 1.41, 95% confidence interval (CI): 1.11–1.78, *p* = 0.004). RCS analysis indicated a positive linear relationship between PHR and diabetes risk (P for nonlinearity = 0.339). Subgroup analyses showed that the association was generally consistent across most subgroups. Sensitivity analyses, including use of a composite diabetes definition (Q4 vs Q1: HR = 1.30, 95% CI: 1.09–1.56) and multiple imputation, yielded consistent findings.

**Conclusions:**

Elevated PHR levels were associated with an increased risk of incident diabetes among Chinese middle-aged and older adults. Component-specific analyses suggested that this association may be driven predominantly by HDL-C. PHR may represent a simple and routinely available candidate biomarker associated with incident diabetes risk; however, whether it provides information beyond HDL-C alone requires further investigation.

**Clinical trial registration:**

Not applicable.

**Supplementary information:**

The online version contains supplementary material available at https://doi.org/10.1186/s12902-026-02627-x.

## Background

Diabetes mellitus has become one of the most critical global public health challenges. According to the International Diabetes Federation, approximately 589 million adults were living with diabetes in 2024, and this number is projected to reach 853 million by 2050 [1]. China carries an exceptionally heavy disease burden, with more than 233 million adults affected in 2023 - a 163% increase since 2005 [2, 3]. Diabetes significantly increases the risks of cardiovascular disease, renal failure, and premature mortality [4, 5], and its annual global economic cost has exceeded US$1.3 trillion [6]. Therefore, identifying simple, cost-effective, and reliable biomarkers associated with diabetes risk is important for addressing this escalating societal and medical burden.

The pathogenesis of diabetes mellitus is closely associated with chronic low-grade inflammation and systemic metabolic disturbance [7, 8]. Persistent inflammatory status is often accompanied by elevated platelet count, enhanced platelet activation, and increased release of inflammatory mediators [9]. Platelets participate in inflammatory responses by aggregating and releasing multiple pro-inflammatory cytokines, including CD40 ligand and P-selectin, which may accelerate insulin resistance and pancreatic β-cell dysfunction [9, 10]. Studies have shown that increased mean platelet volume is independently associated with a higher risk of incident diabetes [11].

In addition, high-density lipoprotein cholesterol (HDL-C) levels are closely associated with the risk of diabetes mellitus and related metabolic disorders [12, 13]. Large cohort studies have demonstrated a negative linear relationship between HDL-C levels and incident diabetes risk, with lower HDL-C levels associated with a higher diabetes incidence [12]. During reverse cholesterol transport, HDL-C facilitates free cholesterol efflux from peripheral tissues while exerting anti-inflammatory, antioxidant, and anti-thrombotic effects, thereby protecting pancreatic β-cell function and improving insulin sensitivity [13, 14]. Given the opposing roles of platelets and HDL-C in inflammation and metabolic homeostasis, PHR, which integrates both pro-thrombotic/inflammatory and protective lipid profiles, may provide an integrated marker associated with diabetes risk.

Recent studies have linked elevated PHR to a range of adverse health outcomes, including cardiovascular disease, stroke, non-alcoholic fatty liver disease, and heart failure [15–18]. Alshuweishi et al. further reported that PHR could predict type 2 diabetes mellitus in obese patients [19]. A cross-sectional study based on NHANES data also demonstrated a positive association between PHR and prevalent diabetes or prediabetes [20]. However, prospective population-based evidence regarding the association between PHR and incident diabetes remains limited. To address this gap, the present study was conducted using data from the China Health and Retirement Longitudinal Study (CHARLS) to investigate the relationship between PHR and the risk of incident diabetes.

## Methods

### Data source and study population

The data used in this study were obtained from CHARLS conducted between 2011 and 2020. CHARLS is a nationally representative longitudinal survey of adults aged 45 years and older in China [21]. Participants were recruited using a multi-stage probability sampling strategy covering 28 provinces, 150 counties, and 450 rural or urban communities across China. The baseline survey was carried out from June 2011 to March 2012, with a total of 17,705 participants enrolled. Follow-up surveys were conducted approximately every two years. At each follow-up wave, standardized information was collected, including demographics, socioeconomic status, health status, physical examinations, and biological sample measurements. The CHARLS study was conducted in accordance with the Declaration of Helsinki and was approved by the Biomedical Ethics Review Committee of Peking University (IRB00001052-11015). All participants provided written informed consent prior to enrollment.

**Fig. 1:**
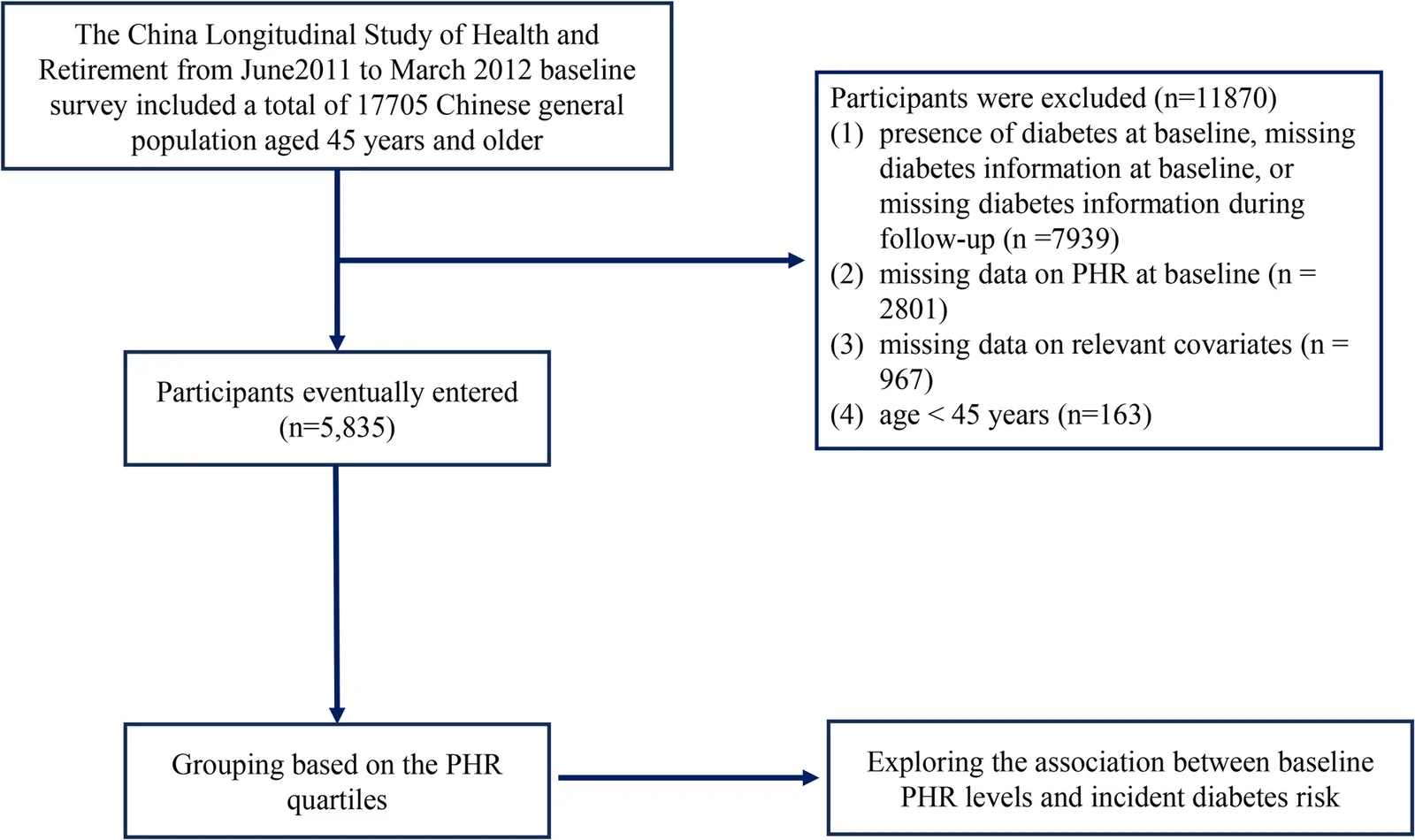
Flowchart of participant selection

Our study included participants who completed the baseline survey (2011–2012) and were followed up in 2013, 2015, 2018, and 2020. Participants without diabetes at baseline were eligible for inclusion. Exclusion criteria were as follows: (1) presence of diabetes at baseline, missing baseline diabetes status, or missing diabetes information during follow-up (*n* = 7939); (2) missing data on PHR at baseline (*n* = 2801); (3) missing data on relevant covariates (*n* = 967); and (4) age < 45 years (*n* = 163). Ultimately, 5,835 participants were included in the final analysis (Fig. 1) and were divided into four subgroups based on baseline PHR quartiles.

### Definition of variables and outcome

In this study, PHR was calculated as platelet count (10^9/L) divided by HDL-C level (mg/dL). Incident diabetes was defined as self-reported physician-diagnosed diabetes during the follow-up period. The primary outcome was incident diabetes.

Covariates collected at baseline included the following: (1) sociodemographic characteristics: age, gender (male/female), residence (urban/rural), education level (less than high school/high school and above); (2) lifestyle factors: smoking status (never/former/current), alcohol consumption (never/former/current); (3) anthropometric measurements: body mass index (BMI); (4) laboratory parameters: platelet count (PLT), HDL-C, low-density lipoprotein cholesterol (LDL-C), total cholesterol (TC), triglycerides (TG), serum creatinine (Scr); and (5) comorbidities: hypertension (self-reported physician-diagnosed) and heart disease (self-reported physician-diagnosed heart problems including coronary heart disease, angina, myocardial infarction, heart failure, and other heart conditions).

### Statistical analysis

Continuous variables were presented as mean ± standard deviation (SD) or median with interquartile range (IQR), depending on data distribution. Categorical variables were summarized as frequencies and percentages. Baseline characteristics across PHR quartiles were compared using ANOVA or the Kruskal-Wallis H test for continuous variables and the chi-squared test for categorical variables. Kaplan-Meier curves were constructed to estimate the cumulative incidence of incident diabetes by PHR quartile, and differences between curves were assessed using the log-rank test. Cox proportional hazards regression models were used to estimate hazard ratios (HRs) and 95% confidence intervals (CIs) for the association between PHR and incident diabetes. Three models were established: Model 1 (unadjusted), Model 2 (adjusted for age and gender), and Model 3 (fully adjusted for age, gender, residence, education, smoking, alcohol consumption, BMI, LDL-C, TG, Scr, hypertension, and heart disease). In additional component-specific analyses, platelet count and HDL-C were separately examined in Cox proportional hazards regression models using the same modeling strategy as those applied to PHR. The proportional hazards assumption was tested using Schoenfeld residuals. Restricted cubic spline (RCS) regression was performed to explore the dose-response relationship between PHR and diabetes risk, with the same covariate adjustment as in the three Cox models. Four knots were placed at the 5th, 35th, 65th, and 95th percentiles of the PHR distribution, with the median PHR value used as the reference. Subgroup analyses were conducted to assess the robustness of the findings. The likelihood ratio test was used to examine interaction effects between PHR and each stratification variable.

### Sensitivity analyses

Several sensitivity analyses were conducted to assess the robustness of the findings. First, an alternative composite definition of incident diabetes was used, defined by fasting blood glucose ≥ 7.0 mmol/L, HbA1c (glycated hemoglobin A1c) ≥6.5%, self-reported physician diagnosis, or current use of antidiabetic medication. For this sensitivity analysis, participants meeting any of these composite diabetes criteria at baseline were excluded to ensure that only incident diabetes cases were identified during follow-up. In this composite-outcome sensitivity analysis, baseline fasting blood glucose, HbA1c, and physical activity were additionally included in the fully adjusted Cox model. In an additional sensitivity analysis, the primary self-reported incident diabetes outcome was retained, while baseline fasting blood glucose and HbA1c were additionally included in the fully adjusted Cox model. Second, we assessed whether incident diabetes occurred within the first 2 years of follow-up as a check for potential reverse causation. Third, participants with extreme platelet count or HDL-C values (below the 10th percentile or above the 90th percentile of their respective distributions) were excluded. Fourth, analyses were repeated using log-transformed PHR and standardized PHR per 1-SD increase.

To assess the potential impact of missing data and selection bias, available baseline characteristics were descriptively compared between participants included in the primary analysis and otherwise eligible participants excluded because of missing data. Multiple imputation was also performed for missing covariate data (excluding the outcome variable) using five imputed datasets; Cox regression estimates were pooled using Rubin’s rules.

All statistical analyses were performed using R software (version 4.3.0) and STATA (version 16.0). A two-sided *p*-value < 0.05 was considered statistically significant.

## Results

### Baseline characteristics

A total of 5835 participants were enrolled, with a median follow-up of 116 months. The cohort comprised 2624 males (44.97%) and 3211 females (55.03%), with a mean age of 57.98 ± 8.55 years. Participants were stratified into four groups based on PHR quartiles: Q1 (*n* = 1459, PHR < 3.01), Q2 (*n* = 1460, 3.01 ≤ PHR <4.11), Q3 (*n* = 1458, 4.11 ≤ PHR <5.51), and Q4 (*n* = 1458, PHR ≥ 5.51). During follow-up, 669 participants (11.47%) developed incident diabetes.

The cumulative incidence of diabetes during follow-up increased progressively across baseline PHR quartiles (Q1: 8.36%, Q2: 10.0%, Q3: 12.0%, Q4: 15.5%) (Table 1). BMI, TG, PLT, and the prevalence of hypertension and heart disease were significantly elevated with increasing PHR. In contrast, HDL-C levels decreased progressively across PHR quartiles. Gender distribution also differed significantly (*p* = 0.029), with a higher proportion of females in the higher PHR groups. Detailed baseline characteristics are presented in Table 1.

**Table 1:** Baseline characteristics of participants

Variables	Total(N = 5835)	Q1 (PHR < 3.01)	Q2 (3.01 ≤ PHR < 4.11)	Q3 (4.11 ≤ PHR < 5.51)	Q4 (PHR ≥ 5.51)	P
		(N = 1459)	(N = 1460)	(N = 1458)	(N = 1458)	
Age,Mean±SD	57.98 ± 8.55	59.21 ± 8.81	58.06 ± 8.37	57.28 ± 8.37	57.38 ± 8.51	0.152
Gender, n(%)						0.029
Male	2624(44.97)	702(48.12)	651(44.59)	648(44.44)	623(42.73)	
Female	3211(55.03)	757(51.88)	809(55.41)	810(55.56)	835(57.27)	
Residence, n(%)						0.437
Rural	5066(86.82)	1277(87.53)	1272(87.12)	1269(87.04)	1248(85.6)	
Urban	769(13.18)	182(12.47)	188(12.88)	189(12.96)	210(14.4)	
Education, n(%)						0.06
Less than high school	5282(90.52)	1341(91.91)	1318(90.27)	1325(90.88)	1298(89.03)	
High school and above	553(9.48)	118(8.09)	142(9.73)	133(9.12)	160(10.97)	
Hypertension, n(%)						<0.001
Yes	1276(21.87)	284(19.47)	274(18.77)	337(23.11)	381(26.13)	
No	4559(78.13)	1175(80.53)	1186(81.23)	1121(76.89)	1077(73.87)	
Heart disease, n (%)						0.028
Yes	593(10.16)	140(9.6)	127(8.7)	152(10.43)	174(11.93)	
No	5242(89.84)	1319(90.4)	1333(91.3)	1306(89.57)	1284(88.07)	
Incident diabetes, n (%)						<0.001
Yes	669(11.47)	122(8.36)	146(10)	175(12)	226(15.5)	
No	5166(88.53)	1337(91.64)	1314(90)	1283(88)	1232(84.5)	
Smoke, n (%)						0.146
Current	1752(30.03)	473(32.42)	439(30.07)	437(29.97)	403(27.64)	
Never	3638(62.35)	883(60.52)	917(62.81)	901(61.80)	937(64.27)	
Former	445(7.63)	103(7.06)	104(7.12)	120(8.23)	118(8.09)	
Alcohol consumption,n(%)						0.423
Current	1469(25.18)	364(24.95)	365(25)	359(24.62)	381(26.13)	
Never	4041(69.25)	1004(68.81)	1011(69.25)	1013(69.48)	1013(69.48)	
Former	325(5.57)	91(6.24)	84(5.75)	86(5.90)	64(4.39)	
BMI(kg/m2),M(Q1,Q3)	23.05(20.81,25.59)	21.93(20.03,24.34)	22.59(20.54,24.73)	23.46(21.25,26.08)	24.37(21.93,27.03)	<0.001
TG(mg/dl),M(Q1,Q3)	102.66(74.34,148.68)	82.31(63.72,113.28)	93.81(69.92,131.87)	109.30(78.77,153.11)	138.51(100.01,206.21)	<0.001
PLT(×109/L),Mean±SD	212.93 ± 73.22	144.14 ± 43.65	195.06 ± 44.13	230.58 ± 48.23	282.02 ± 71.68	<0.001
HDL-C(mg/dl),Mean±SD	51.85 ± 15.07	64.33 ± 15.59	54.98 ± 12.32	48.83 ± 10.08	39.23 ± 8.84	<0.001
TC(mg/dl),Mean±SD	193.64 ± 37.81	195.21 ± 35.14	194.45 ± 37.54	193.83 ± 37.96	191.07 ± 40.34	<0.001
Scr(mg/dl),Mean±SD	0.77 ± 0.18	0.78 ± 0.18	0.77 ± 0.18	0.77 ± 0.18	0.77 ± 0.18	0.353
LDL-C(mg/dl),Mean±SD	116.84 ± 34.50	114.00 ± 31.52	118.41 ± 34.47	119.77 ± 34.13	115.18 ± 37.36	<0.001
PHR,M(Q1,Q3)	4.11(3.00,5.51)	2.35(1.93,2.72)	3.56(3.29,3.83)	4.68(4.39,5.07)	6.75(6.05,8.00)	<0.001

SD, standard deviation; M, median; PHR, platelet-to-high-density lipoprotein cholesterol ratio; BMI, body mass index; TG, triglycerides; PLT,platelet; HDL-C, high-density lipoprotein cholesterol; TC, total cholesterol; Scr, serum creatinine; LDL-C, low-density lipoprotein cholesterol

### Association between PHR and incident diabetes risk

**Fig. 2:**
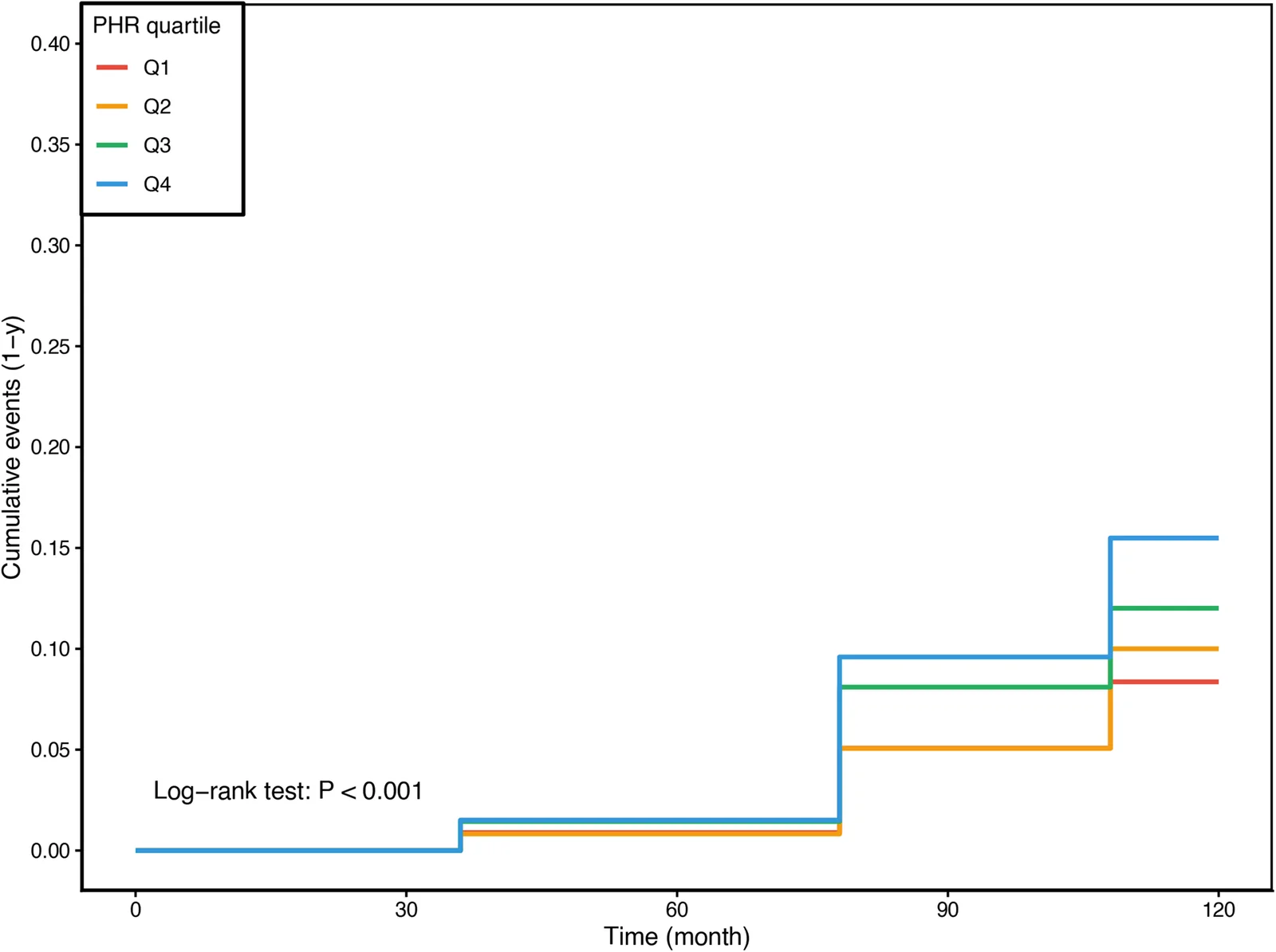
Kaplan-Meier cumulative risk curves for incident diabetes by PHR. PHR, platelets to high density lipoprotein cholesterol ratio

Kaplan-Meier survival curves showed that participants in higher PHR quartiles had a significantly higher cumulative incidence of diabetes than those in lower quartiles (log-rank *p* < 0.001) (Fig. 2).

Cox proportional hazards regression analysis revealed that higher PHR quartiles were significantly associated with an increased risk of diabetes compared with the lowest quartile across all three models (Table 2). In the fully adjusted Model 3, compared with participants in Q1, those in Q4 had a 41% increased risk of diabetes (HR = 1.41, 95% CI: 1.11–1.78, *p* = 0.004). When PHR was analyzed as a continuous variable, each 1-unit increase in PHR was associated with a 6% increased risk of diabetes (HR = 1.06, 95% CI: 1.02–1.09, *p* = 0.001). The trend test confirmed a consistent increase in diabetes risk with rising PHR values (P for trend = 0.002).

**Table 2:** Association between PHR and incident diabetes risk in different Cox proportional hazards models

Exposure	Model 1	P	Model 2	P	Model 3	P
	HR(95% CI)		HR(95% CI)		HR(95% CI)	
PHR	1.11 (1.08, 1.14)	<0.001	1.11 (1.08, 1.14)	<0.001	1.06 (1.02, 1.09)	0.001
PHR quartile						
Q1	1.0		1.0		1.0	
Q2	1.20 (0.94, 1.53)	0.137	1.21 (0.95, 1.53)	0.128	1.12 (0.88, 1.43)	0.345
Q3	1.47 (1.17, 1.85)	0.001	1.48 (1.18, 1.87)	0.001	1.27 (1.00, 1.61)	0.046
Q4	1.93 (1.55, 2.40)	<0.001	1.94 (1.56, 2.42)	<0.001	1.41 (1.11, 1.78)	0.004
PHR for trend	1.16 (1.11, 1.21)	<0.001	1.16 (1.11, 1.22)	<0.001	1.08 (1.03, 1.13)	0.002

Model 1: not adjusted

Model 2: adjusted for age and gender

Model 3: adjusted for age, gender, residence, education, smoking, alcohol consumption, BMI, TG, LDL-C, Scr, hypertension, and heart disease

PHR, platelet-to-high-density lipoprotein cholesterol ratio; HR, hazard ratio; CI, confidence interval; BMI, body mass index; TG, triglycerides; Scr, serum creatinine; LDL-C, low-density lipoprotein cholesterol

**Fig. 3:**
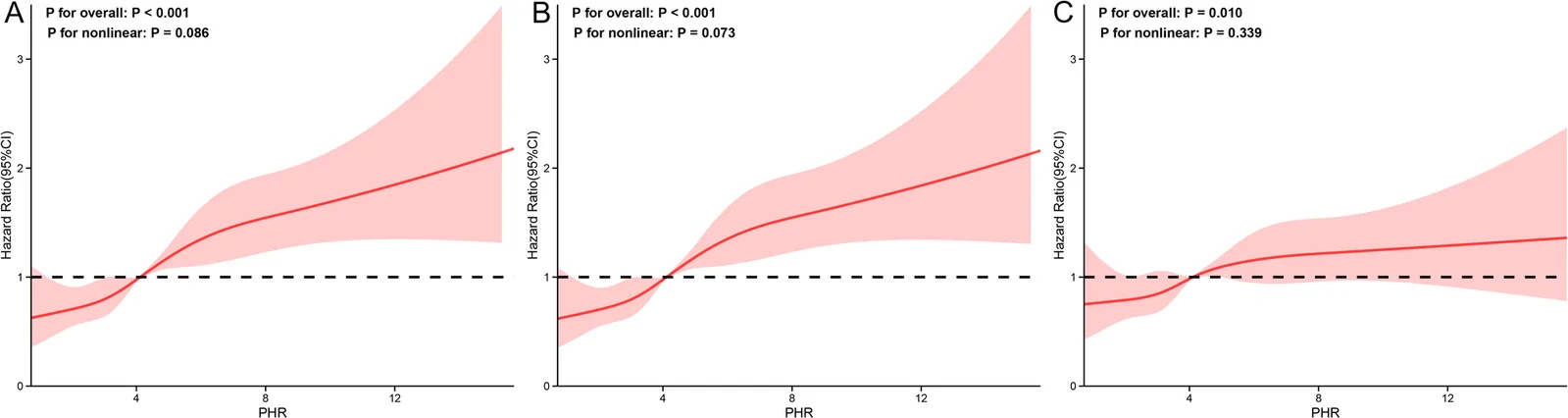
Restricted cubic spline analysis of PHR and incident diabetes. (**A**: not adjusted. **B**: adjusted for age and gender. **C**: adjusted for age, gender, residence, education, smoking, alcohol consumption, BMI, TG, LDL-C, Scr, hypertension, and heart disease.) CI, confidence interval; PHR, platelets to high density lipoprotein cholesterol ratio; BMI, body mass index; TG, triglycerides; Scr, serum creatinine; LDL-C, low-density lipoprotein cholesterol

RCS regression analysis revealed a positive linear association between PHR and diabetes risk in Model 1 (unadjusted, P for overall < 0.001, P for nonlinearity = 0.086), Model 2 (age- and gender-adjusted, P for overall < 0.001, P for nonlinearity = 0.073), and Model 3 (fully adjusted, P for overall = 0.010, P for nonlinearity = 0.339) (Fig. 3).

### Component-specific analyses

In additional component-specific analyses, platelet count alone was not significantly associated with incident diabetes in the fully adjusted model, whereas higher HDL-C was significantly associated with a lower risk of incident diabetes (Q4 vs Q1: PLT, HR = 1.13, 95% CI: 0.91–1.39, *p* = 0.267; HDL-C, HR = 0.54, 95% CI: 0.42–0.70, *p* < 0.001) (Supplementary Table S1).

### Sensitivity analyses

Using the composite definition of incident diabetes, 1129 incident cases were identified, compared with 669 cases in the primary analysis. In the composite-outcome model with additional adjustment for baseline fasting blood glucose, HbA1c, and physical activity, participants in Q4 remained at higher risk of incident diabetes than those in Q1 (HR = 1.30, 95% CI: 1.09–1.56, *p* = 0.004) (Supplementary Table S2). No incident diabetes cases occurred within the first 2 years of follow-up; therefore, exclusion of early events did not alter the analytic sample or the results. Analyses excluding extreme platelet/HDL-C values and analyses using log-transformed or standardized PHR were generally consistent with the primary findings (Supplementary Tables S3–S5).

Available baseline characteristics were broadly comparable between included participants and otherwise eligible participants excluded because of missing data (Supplementary Table S6). In the multiple-imputation analysis, the pooled HR for Q4 versus Q1 was 1.48 (95% CI: 1.18–1.86, *p* = 0.001), consistent with the complete-case analysis (Supplementary Table S7).

When the primary self-reported diabetes outcome was retained and baseline fasting blood glucose and HbA1c were additionally included in the fully adjusted model, the association was attenuated but remained statistically significant (Q4 vs Q1: HR = 1.30, 95% CI: 1.02–1.64, *p* = 0.031; continuous PHR: HR = 1.04, 95% CI: 1.01–1.08, *p* = 0.016) (Supplementary Table S8).

### Subgroup analysis

**Fig. 4:**
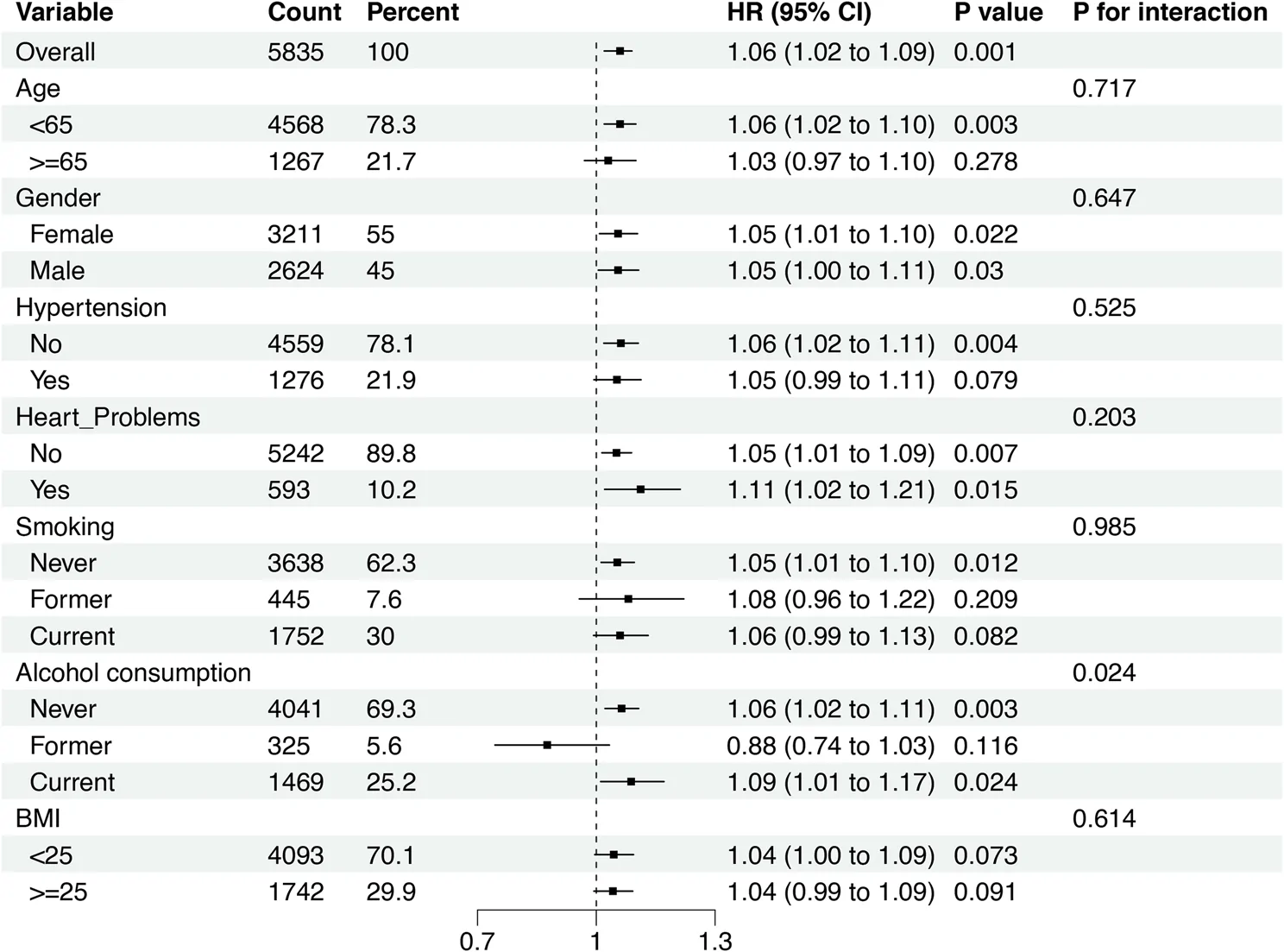
Subgroup analysis. HR, hazard ratio; CI, confidence interval; BMI, body mass index

Subgroup analyses demonstrated that the positive association between PHR and diabetes risk was generally consistent across most subgroups (Fig. 4). A nominal interaction was observed for alcohol consumption (P for interaction = 0.024). No significant interactions were found for age, gender, hypertension, heart disease, smoking, or BMI.

## Discussion

Prospective evidence regarding the association between PHR and incident diabetes remains limited, particularly among Chinese middle-aged and older adults. In this study, we found a positive association between PHR and incident diabetes, which remained statistically significant after adjusting for a wide range of covariates. In the fully adjusted model, each 1-unit increase in PHR was associated with a 6% higher risk of diabetes (HR = 1.06, 95% CI: 1.02–1.09, *p* = 0.001). Participants in the highest PHR quartile exhibited a 41% increased diabetes risk compared with those in the lowest quartile (HR = 1.41, 95% CI: 1.11–1.78, *p* = 0.004). Furthermore, RCS regression revealed a linear trend, indicating a gradual increase in diabetes risk with rising PHR values. The findings were generally consistent across subgroup and multiple sensitivity analyses. In addition, when the primary outcome definition was retained, further adjustment for baseline fasting blood glucose and HbA1c attenuated but did not eliminate the association, suggesting that baseline glycaemic status may partly account for, but does not fully explain, the observed association. These findings support the potential of PHR as a candidate biomarker associated with incident diabetes risk.

In recent years, PHR has emerged as a novel biomarker in metabolic disease research. Jialal et al. first proposed PHR as a valid indicator of metabolic syndrome, demonstrating that individuals with metabolic syndrome had significantly higher PHR levels than healthy controls [22]. Our finding of a positive association between PHR and incident diabetes aligns with previous research. Alshuweishi et al. reported that PHR could predict type 2 diabetes in obese patients via a retrospective study [19]. A recent cross-sectional analysis of NHANES data by Chen et al. identified a positive association between PHR and diabetes/prediabetes in US adults, with participants in the highest PHR quartile having an odds ratio of 2.55 compared with the lowest quartile [20]. That study also detected an inflection point at PHR = 4.55, suggesting a potential threshold effect. Whereas our study found a linear relationship without a clear threshold, these discrepancies may be attributed to differences in study design (cross-sectional vs. prospective cohort), population characteristics (US vs. Chinese middle-aged and older adults), and outcome definition (prevalent vs. incident diabetes).

Recent longitudinal evidence has also linked baseline and cumulative PHR with cardiovascular disease risk [23]. Beyond PHR, longitudinal studies have evaluated HDL-C-containing lipid ratios and other composite cardiometabolic indices, including the atherogenic index of plasma, the triglyceride-glucose and non-HDL-to-HDL cholesterol ratio (TyG-NHHR), and the modified cardiometabolic index, in relation to stroke, cardiometabolic multimorbidity, and incident chronic diseases [24–26]. Our study adds diabetes-specific prospective evidence by examining the association between PHR and incident diabetes in a large population-based cohort.

It should be noted that our additional component-specific analyses suggested that the association between PHR and incident diabetes may be driven predominantly by the HDL-C component, whereas platelet count alone was not significantly associated with incident diabetes in this cohort. This finding does not exclude a potential role of platelet-related inflammatory or thrombotic processes, as platelet count does not directly reflect platelet activation or function. Nevertheless, PHR remained significantly associated with incident diabetes, although further studies are needed to determine whether it provides information beyond HDL-C alone. Several biological mechanisms may underlie the observed association between PHR and diabetes. First, chronic low-grade inflammation is a well-recognized driver of insulin resistance and pancreatic β-cell dysfunction [7, 27]. Platelets are important mediators of inflammatory responses. Upon activation, they release pro-inflammatory mediators, including CD40 ligand, P-selectin, RANTES, and platelet-derived extracellular vesicles, which may contribute to endothelial activation, leukocyte recruitment, and systemic inflammation [9, 10]. Second, HDL-C exerts anti-inflammatory, antioxidant, and anti-thrombotic effects that protect against diabetes development [12, 13]. HDL-C also directly modulates glucose metabolism by stimulating insulin secretion from pancreatic β-cells, enhancing glucose uptake in skeletal muscle and adipose tissue, and protecting β-cells from apoptosis [14, 28]. Lower HDL-C levels, reflected by elevated PHR, may compromise these protective functions, thereby increasing diabetes susceptibility. Third, platelet activation and HDL-C dysfunction may jointly contribute to diabetes pathogenesis. HDL-C can inhibit platelet activation through several mechanisms, including prevention of intraplatelet cholesterol overload and stimulation of endothelial prostacyclin and nitric oxide production [29]. Thus, although the present component-specific analysis suggests that HDL-C is the predominant contributor to the observed PHR-diabetes association, PHR may still reflect combined platelet-related and HDL-C-related biological information.

Additionally, our subgroup analyses demonstrated that the positive association between PHR and incident diabetes risk was generally consistent across most demographic and clinical subgroups. The nominal interaction observed for alcohol consumption (P for interaction = 0.024) should be interpreted cautiously because multiple subgroup tests were performed and it may represent a chance finding. The nonsignificant estimate among former drinkers may also be influenced by health-related drinking cessation (the “sick quitter” phenomenon). These exploratory findings require confirmation in future studies.

### Strengths

This study has several notable strengths. First, it provides prospective evidence regarding the association between PHR and incident diabetes in China, where such evidence remains limited. Second, we utilized a large, nationally representative cohort with a long follow-up period, enhancing the generalizability and statistical power of our findings. Third, the consistency of the association across multiple sensitivity analyses addressing outcome definition, missing data, extreme values, and PHR parameterization further supports the robustness of the findings.

### Limitations

Several limitations should be acknowledged. First, self-reported diabetes in the primary analysis may underestimate undiagnosed cases, although the findings were consistent using a composite biochemical and clinical definition. Second, PHR was measured only at baseline and the cohort was limited to Chinese adults aged 45 years and older, restricting assessment of temporal changes and generalizability. Third, residual confounding and selection bias remain possible despite expanded covariate adjustment and multiple imputation. Fourth, competing-risk analysis for death was not feasible because precise timing of death was not consistently available. Finally, this study assessed association rather than prediction; predictive performance, including discrimination, calibration, and threshold performance, and incremental value were not formally assessed and require dedicated validation.

## Conclusions

In conclusion, this large prospective cohort study demonstrates that elevated PHR is linearly associated with an increased risk of incident diabetes in Chinese middle-aged and older adults. Component-specific analyses suggest that this association may be driven predominantly by HDL-C, whereas platelet count alone was not significantly associated with incident diabetes. The association was generally consistent across subgroup and sensitivity analyses. PHR may represent a convenient and routinely available candidate biomarker associated with incident diabetes risk; however, whether it provides information beyond HDL-C alone requires further investigation.

## Electronic supplementary material

Below is the link to the electronic supplementary material.

Supplementary Material 1

Supplementary Material 2

Supplementary Material 3

Supplementary Material 4

Supplementary Material 5

Supplementary Material 6

Supplementary Material 7

Supplementary Material 8

## Data Availability

The CHARLS data are publicly available athttp://charls.pku.edu.cn/. The authors confirm that they had no special access privileges to the data.

## Abbreviations

BMI: Body mass index
CHARLS: China health and retirement longitudinal study
CI: Confidence interval
HDL-C: High-density lipoprotein cholesterol
HR: Hazard ratio
IQR: Interquartile range
LDL-C: Low-density lipoprotein cholesterol
PHR: Platelet-to-high-density lipoprotein cholesterol ratio
PLT: Platelet count
RCS: Restricted cubic spline
Scr: Serum creatinine
SD: Standard deviation
TC: Total cholesterol
TG: Triglycerides
HbA1c: Glycated hemoglobin A1c
