# Supplementary material for: Association between platelet-to-high-density lipoprotein cholesterol ratio and incident diabetes risk: a national representative cohort study based on CHARLS
Source: BMC Endocr Disord. 2026 Oct 12;26:347. doi: 10.1186/s12902-026-02627-x (PMC13733031; doi:10.1186/s12902-026-02627-x)
Supplement: Supplementary file 1 [file 12902_2026_2627_MOESM1_ESM.docx]

**Table S1. Association of PLT and HDL-C With Incident Diabetes**

| Exposure | Model 1 | | Model 2 | | Model 3 | |
| --- | --- | --- | --- | --- | --- | --- |
|  | HR  (95% CI) | P | HR  (95% CI) | P | HR  (95% CI) | P |
| PLT | 1.00 (1.00, 1.00) | 0.133 | 1.00 (1.00, 1.00) | 0.146 | 1.00 (1.00, 1.00) | 0.321 |
| PLT quartile |  |  |  |  |  |  |
| Q1 | 1.0 |  | 1.0 |  | 1.0 |  |
| Q2 | 1.02 (0.83, 1.27) | 0.836 | 1.02 (0.82, 1.27) | 0.846 | 1.02 (0.82, 1.27) | 0.845 |
| Q3 | 0.91 (0.73, 1.14) | 0.417 | 0.91 (0.73, 1.14) | 0.400 | 0.84 (0.67, 1.05) | 0.126 |
| Q4 | 1.17 (0.95, 1.45) | 0.131 | 1.17 (0.95, 1.44) | 0.144 | 1.13 (0.91, 1.39) | 0.267 |
| PLT for trend | 1.00 (1.00, 1.00) | 0.206 | 1.00 (1.00, 1.00) | 0.225 | 1.00 (1.00, 1.00) | 0.478 |
| HDL-C | 0.98 (0.97, 0.98) | <0.001 | 0.97 (0.97, 0.98) | <0.001 | 0.98 (0.98, 0.99) | <0.001 |
| HDL-C quartile |  |  |  |  |  |  |
| Q1 | 1.0 |  | 1.0 |  | 1.0 |  |
| Q2 | 0.67 (0.55, 0.81) | <0.001 | 0.66 (0.55, 0.81) | <0.001 | 0.77 (0.63, 0.95) | 0.014 |
| Q3 | 0.54 (0.44, 0.67) | <0.001 | 0.53 (0.43, 0.65) | <0.001 | 0.69 (0.55, 0.86) | 0.001 |
| Q4 | 0.39 (0.31, 0.49) | <0.001 | 0.38 (0.30, 0.48) | <0.001 | 0.54 (0.42, 0.70) | <0.001 |
| HDL-C for trend | 0.97 (0.97, 0.98) | <0.001 | 0.97 (0.96, 0.98) | <0.001 | 0.98 (0.97, 0.99) | <0.001 |

Model 1: not adjusted.

Model 2: adjusted for age and gender.

Model 3: adjusted for age, gender, residence, education, smoking, alcohol consumption, BMI, TG, LDL-C, Scr, hypertension, and heart disease.

HR, hazard ratio; CI, confidence interval;

BMI, body mass index; TG, triglycerides; Scr, serum creatinine; LDL-C, low-density lipoprotein cholesterol; HDL-C, high-density lipoprotein cholesterol

**Table S2. Association Between PHR and Incident Diabetes Using a Composite Definition of Diabetes**

| Exposure | Model 1 |  | Model 2 |  | Model 3 |  |
| --- | --- | --- | --- | --- | --- | --- |
|  | HR(95% CI) | P | HR(95% CI) | P | HR(95% CI) | P |
| PHR | 1.11 (1.09, 1.13) | <0.001 | 1.11 (1.09, 1.14) | <0.001 | 1.05 (1.03, 1.08) | <0.001 |
| PHR quartile |  |  |  |  |  |  |
| Q1 | 1.0 |  | 1.0 |  | 1.0 |  |
| Q2 | 1.14 (0.95, 1.37) | 0.161 | 1.16 (0.96, 1.39) | 0.122 | 1.11 (0.93, 1.34) | 0.248 |
| Q3 | 1.36 (1.14, 1.62) | 0.001 | 1.39 (1.16, 1.66) | <0.001 | 1.23 (1.03, 1.48) | 0.022 |
| Q4 | 1.86 (1.57, 2.19) | <0.001 | 1.90 (1.61, 2.24) | <0.001 | 1.30 (1.09, 1.56) | 0.004 |
| PHR for trend | 1.15 (1.11, 1.20) | <0.001 | 1.16 (1.12, 1.20) | <0.001 | 1.06 (1.02, 1.10) | 0.003 |

In this sensitivity analysis, diabetes was defined as fasting blood glucose ≥7.0 mmol/L, HbA1c ≥6.5%, self-reported physician-diagnosed diabetes, or current use of antidiabetic medication. Participants meeting any of these criteria at baseline were excluded.

Model 1: not adjusted.

Model 2: adjusted for age and gender.

Model 3: adjusted for age, gender, residence, education, smoking, alcohol consumption, BMI, TG, LDL-C, Scr, fasting blood glucose, HbA1c, physical activity, hypertension, and heart disease.

PHR, platelet-to-high-density lipoprotein cholesterol ratio; HR, hazard ratio; CI, confidence interval;

BMI, body mass index; TG, triglycerides; Scr, serum creatinine; LDL-C, low-density lipoprotein cholesterol; HbA1c, glycated hemoglobin A1c.

**Table S3. Association Between Log-Transformed PHR and Incident Diabetes**

| Exposure | Model 1 |  | Model 2 |  | Model 3 |  |
| --- | --- | --- | --- | --- | --- | --- |
|  | HR(95% CI) | P | HR(95% CI) | P | HR(95% CI) | P |
| Log-transformed PHR | 1.79 (1.52, 2.12) | <0.001 | 1.80 (1.53, 2.13) | <0.001 | 1.37 (1.15, 1.63) | 0.001 |
| PHR quartile |  |  |  |  |  |  |
| Q1 | 1.0 |  | 1.0 |  | 1.0 |  |
| Q2 | 1.20 (0.94, 1.53) | 0.137 | 1.21 (0.95, 1.53) | 0.128 | 1.12 (0.88, 1.43) | 0.345 |
| Q3 | 1.47 (1.17, 1.85) | 0.001 | 1.48 (1.18, 1.87) | 0.001 | 1.27 (1.00, 1.61) | 0.046 |
| Q4 | 1.93 (1.55, 2.40) | <0.001 | 1.94 (1.56, 2.42) | <0.001 | 1.41 (1.11, 1.78) | 0.004 |
| PHR for trend | 1.90 (1.55, 2.32) | <0.001 | 1.91 (1.56, 2.34) | <0.001 | 1.39 (1.13, 1.72) | 0.002 |

Model 1: not adjusted.

Model 2: adjusted for age and gender.

Model 3: adjusted for age, gender, residence, education, smoking, alcohol consumption, BMI, TG, LDL-C, Scr, hypertension, and heart disease.

PHR, platelet-to-high-density lipoprotein cholesterol ratio; HR, hazard ratio; CI, confidence interval;

BMI, body mass index; TG, triglycerides; Scr, serum creatinine; LDL-C, low-density lipoprotein cholesterol.

**Table S4. Association Between Standardized PHR (per 1-SD Increase) and Incident Diabetes**

PHR was standardized and analyzed per 1-SD increase

| Exposure | Model 1 |  | Model 2 |  | Model 3 |  |
| --- | --- | --- | --- | --- | --- | --- |
|  | HR(95% CI) | P | HR(95% CI) | P | HR(95% CI) | P |
| PHR (per 1-SD increase) | 1.25 (1.18, 1.33) | <0.001 | 1.25 (1.18, 1.33) | <0.001 | 1.13 (1.05, 1.21) | 0.001 |
| PHR quartile |  |  |  |  |  |  |
| Q1 | 1.0 |  | 1.0 |  | 1.0 |  |
| Q2 | 1.20 (0.94, 1.53) | 0.137 | 1.21 (0.95, 1.53) | 0.128 | 1.12 (0.88, 1.43) | 0.345 |
| Q3 | 1.47 (1.17, 1.85) | 0.001 | 1.48 (1.18, 1.87) | 0.001 | 1.27 (1.00, 1.61) | 0.046 |
| Q4 | 1.93 (1.55, 2.40) | <0.001 | 1.94 (1.56, 2.42) | <0.001 | 1.41 (1.11, 1.78) | 0.004 |
| PHR for trend | 1.38 (1.25, 1.52) | <0.001 | 1.38 (1.25, 1.53) | <0.001 | 1.18 (1.06, 1.31) | 0.002 |

Model 1: not adjusted.

Model 2: adjusted for age and gender.

Model 3: adjusted for age, gender, residence, education, smoking, alcohol consumption, BMI, TG, LDL-C, Scr, hypertension, and heart disease.

PHR, platelet-to-high-density lipoprotein cholesterol ratio; HR, hazard ratio; CI, confidence interval; SD, standard deviation;

BMI, body mass index; TG, triglycerides; Scr, serum creatinine; LDL-C, low-density lipoprotein cholesterol.

**Table S5. Association Between PHR and Incident Diabetes After Excluding Extreme Platelet or HDL-C Values**

Extreme values were defined as platelet count or HDL-C values below the 10th percentile or above the 90th percentile.

| Exposure | Model 1 |  | Model 2 |  | Model 3 |  |
| --- | --- | --- | --- | --- | --- | --- |
|  | HR(95% CI) | P | HR(95% CI) | P | HR(95% CI) | P |
| PHR | 1.13 (1.09, 1.17) | <0.001 | 1.13 (1.09, 1.17) | <0.001 | 1.06 (1.02, 1.11) | 0.001 |
| PHR quartile |  |  |  |  |  |  |
| Q1 | 1.0 |  | 1.0 |  | 1.0 |  |
| Q2 | 1.18 (0.93, 1.50) | 0.137 | 1.18 (0.93, 1.51) | 0.174 | 1.10 (0.86, 1.40) | 0.437 |
| Q3 | 1.45 (1.15, 1.82) | 0.001 | 1.46 (1.15, 1.84) | 0.002 | 1.24 (0.98, 1.58) | 0.070 |
| Q4 | 1.86 (1.49, 2.33) | <0.001 | 1.88 (1.50, 2.35) | <0.001 | 1.38 (1.09, 1.75) | 0.008 |
| PHR for trend | 1.16 (1.10, 1.21) | <0.001 | 1.16 (1.10, 1.21) | <0.001 | 1.08 (1.02, 1.13) | 0.005 |

Model 1: not adjusted.

Model 2: adjusted for age and gender.

Model 3: adjusted for age, gender, residence, education, smoking, alcohol consumption, BMI, TG, LDL-C, Scr, hypertension, and heart disease.

PHR, platelet-to-high-density lipoprotein cholesterol ratio; HR, hazard ratio; CI, confidence interval;

BMI, body mass index; TG, triglycerides; Scr, serum creatinine; LDL-C, low-density lipoprotein cholesterol; HDL-C, high-density lipoprotein cholesterol.

**Table S6. Baseline Characteristics of Included Participants and Otherwise Eligible Participants Excluded Due to Missing Data**

| Characteristic | Included participants N = 5835 | Excluded participants due to missing data N = 3768 |
| --- | --- | --- |
| Age, years, M(Q1,Q3) | 57 (51, 63) | 56 (50, 62) |
| Gender, n(%) |  |  |
| Male | 2624 (44.97%) | 1922 (51.01%) |
| Female | 3211 (55.03%) | 1846 (48.99%) |
| Residence, n(%) |  |  |
| Urban | 769 (13.18%) | 754 (20.01%) |
| Rural | 5066 (86.82%) | 3014 (79.99%) |
| Education, n(%) |  |  |
| Less than high school | 5282 (90.52%) | 3278 (87.0%) |
| High school and above | 553 (9.48%) | 490 (13.0%) |
| Smoke, n (%) |  |  |
| Never | 3638 (62.35%) | 2298 (60.99%) |
| Former | 445 (7.63%) | 264 (7.01%) |
| Current | 1752 (30.02%) | 1206 (32%) |
| Alcohol consumption, n(%) |  |  |
| Never | 4041 (69.25%) | 2562 (67.99%) |
| Former | 325 (5.57%) | 188 (5.0%) |
| Current | 1469 (25.18%) | 1018 (27.01%) |
| Hypertension, n(%) |  |  |
| No | 4559 (78.13%) | 3052 (81.0%) |
| Yes | 1276 (21.87%) | 716 (19.0%) |
| Heart disease, n (%) |  |  |
| No | 5242 (89.84%) | 3429 (91.0%) |
| Yes | 593 (10.16%) | 339 (9.0%) |
| BMI, kg/m2, M(Q1,Q3) | 23.0 (20.8, 25.6) | 23.0 (20.8, 25.5) |
| LDL-C, mg/dL, M(Q1,Q3) | 115 (94, 137) | 114 (92, 137) |
| TG, mg/dL, M(Q1,Q3) | 103 (74, 149) | 104 (74, 154) |
| Scr, mg/dL, M(Q1,Q3) | 0.75 (0.64, 0.87) | 0.77 (0.66, 0.88) |

Excluded participants were otherwise eligible participants without baseline diabetes who were excluded from the primary analysis because of missing data.

M, median; BMI, body mass index; TG, triglycerides; Scr, serum creatinine; LDL-C, low-density lipoprotein cholesterol.

**Table S7. Association Between PHR and Incident Diabetes Using Multiple Imputation**

| Exposure | Model 1 |  | Model 2 |  | Model 3 |  |
| --- | --- | --- | --- | --- | --- | --- |
|  | HR(95% CI) | P | HR(95% CI) | P | HR(95% CI) | P |
| PHR | 1.10 (1.08, 1.13) | <0.001 | 1.10 (1.08, 1.13) | <0.001 | 1.06 (1.03, 1.09) | <0.001 |
| PHR quartile |  |  |  |  |  |  |
| Q1 | 1.0 |  | 1.0 |  | 1.0 |  |
| Q2 | 1.12 (0.82, 1.51) | 0.455 | 1.12 (0.82, 1.52) | 0.441 | 1.08 (0.79, 1.47) | 0.593 |
| Q3 | 1.32 (1.07, 1.64) | 0.011 | 1.33 (1.07, 1.65) | 0.010 | 1.22 (0.98, 1.53) | 0.072 |
| Q4 | 1.81 (1.48, 2.22) | <0.001 | 1.82 (1.49, 2.23) | <0.001 | 1.48 (1.18, 1.86) | 0.001 |
| PHR for trend | 1.15 (1.11, 1.19) | <0.001 | 1.15 (1.11, 1.20) | <0.001 | 1.10 (1.05, 1.14) | <0.001 |

Missing covariate data were imputed using multiple imputation with five imputed datasets. Cox proportional hazards regression models were fitted separately in each imputed dataset, and estimates were pooled according to Rubin’s rules.

Model 1: not adjusted.

Model 2: adjusted for age and gender.

Model 3: adjusted for age, gender, residence, education, smoking, alcohol consumption, BMI, TG, LDL-C, Scr, hypertension, and heart disease.

PHR, platelet to high density lipoprotein cholesterol ratio; HR, hazard ratio; CI, confidence interval;

BMI, body mass index; TG, triglycerides; Scr, serum creatinine; LDL-C, low-density lipoprotein cholesterol.

**Table S8. Association Between PHR and Incident Diabetes With Additional Adjustment for Fasting Blood Glucose and HbA1c**

| Exposure | Model 1 |  | Model 2 |  | Model 3 |  |
| --- | --- | --- | --- | --- | --- | --- |
|  | HR(95% CI) | P | HR(95% CI) | P | HR(95% CI) | P |
| PHR | 1.11 (1.08, 1.14) | <0.001 | 1.11 (1.08, 1.14) | <0.001 | 1.04 (1.01, 1.08) | 0.016 |
| PHR quartile |  |  |  |  |  |  |
| Q1 | 1.0 |  | 1.0 |  | 1.0 |  |
| Q2 | 1.20 (0.94, 1.53) | 0.137 | 1.21 (0.95, 1.53) | 0.128 | 1.16 (0.91, 1.48) | 0.225 |
| Q3 | 1.47 (1.17, 1.85) | 0.001 | 1.48 (1.18, 1.87) | 0.001 | 1.28 (1.01, 1.62) | 0.037 |
| Q4 | 1.93 (1.55, 2.40) | <0.001 | 1.94 (1.56, 2.42) | <0.001 | 1.30 (1.02, 1.64) | 0.031 |
| PHR for trend | 1.16 (1.11, 1.21) | <0.001 | 1.16 (1.11, 1.22) | <0.001 | 1.05 (1.00, 1.11) | 0.037 |

Model 1: not adjusted.

Model 2: adjusted for age and gender.

Model 3: adjusted for age, gender, residence, education, smoking, alcohol consumption, BMI, TG, LDL-C, Scr , hypertension, heart disease, fasting blood glucose, and HbA1c.

PHR, platelet-to-high-density lipoprotein cholesterol ratio; HR, hazard ratio; CI, confidence interval.

BMI, body mass index; TG, triglycerides; Scr, serum creatinine; LDL-C, low-density lipoprotein cholesterol; HbA1c, glycated hemoglobin A1c.
