# Supplementary material for: Association between platelet-to-high-density lipoprotein cholesterol ratio and incident diabetes risk: a national representative cohort study based on CHARLS
Source: BMC Endocr Disord. 2026 Oct 12;26:347. doi: 10.1186/s12902-026-02627-x (PMC13733031; doi:10.1186/s12902-026-02627-x)
Supplement: Supplementary file 2 [file 12902_2026_2627_MOESM2_ESM.docx]

**Table S2. Association Between PHR and Incident Diabetes Using a Composite Definition of Diabetes**

| Exposure | Model 1 |  | Model 2 |  | Model 3 |  |
| --- | --- | --- | --- | --- | --- | --- |
|  | HR(95% CI) | P | HR(95% CI) | P | HR(95% CI) | P |
| PHR | 1.11 (1.09, 1.13) | <0.001 | 1.11 (1.09, 1.14) | <0.001 | 1.05 (1.03, 1.08) | <0.001 |
| PHR quartile |  |  |  |  |  |  |
| Q1 | 1.0 |  | 1.0 |  | 1.0 |  |
| Q2 | 1.14 (0.95, 1.37) | 0.161 | 1.16 (0.96, 1.39) | 0.122 | 1.11 (0.93, 1.34) | 0.248 |
| Q3 | 1.36 (1.14, 1.62) | 0.001 | 1.39 (1.16, 1.66) | <0.001 | 1.23 (1.03, 1.48) | 0.022 |
| Q4 | 1.86 (1.57, 2.19) | <0.001 | 1.90 (1.61, 2.24) | <0.001 | 1.30 (1.09, 1.56) | 0.004 |
| PHR for trend | 1.15 (1.11, 1.20) | <0.001 | 1.16 (1.12, 1.20) | <0.001 | 1.06 (1.02, 1.10) | 0.003 |

In this sensitivity analysis, diabetes was defined as fasting blood glucose ≥7.0 mmol/L, HbA1c ≥6.5%, self-reported physician-diagnosed diabetes, or current use of antidiabetic medication. Participants meeting any of these criteria at baseline were excluded.

Model 1: not adjusted.

Model 2: adjusted for age and gender.

Model 3: adjusted for age, gender, residence, education, smoking, alcohol consumption, BMI, TG, LDL-C, Scr, fasting blood glucose, HbA1c, physical activity, hypertension, and heart disease.

PHR, platelet-to-high-density lipoprotein cholesterol ratio; HR, hazard ratio; CI, confidence interval;

BMI, body mass index; TG, triglycerides; Scr, serum creatinine; LDL-C, low-density lipoprotein cholesterol; HbA1c, glycated hemoglobin A1c.
