# Supplementary material for: Association between platelet-to-high-density lipoprotein cholesterol ratio and incident diabetes risk: a national representative cohort study based on CHARLS
Source: BMC Endocr Disord. 2026 Oct 12;26:347. doi: 10.1186/s12902-026-02627-x (PMC13733031; doi:10.1186/s12902-026-02627-x)
Supplement: Supplementary file 3 [file 12902_2026_2627_MOESM3_ESM.docx]

**Table S3. Association Between Log-Transformed PHR and Incident Diabetes**

| Exposure | Model 1 |  | Model 2 |  | Model 3 |  |
| --- | --- | --- | --- | --- | --- | --- |
|  | HR(95% CI) | P | HR(95% CI) | P | HR(95% CI) | P |
| Log-transformed PHR | 1.79 (1.52, 2.12) | <0.001 | 1.80 (1.53, 2.13) | <0.001 | 1.37 (1.15, 1.63) | 0.001 |
| PHR quartile |  |  |  |  |  |  |
| Q1 | 1.0 |  | 1.0 |  | 1.0 |  |
| Q2 | 1.20 (0.94, 1.53) | 0.137 | 1.21 (0.95, 1.53) | 0.128 | 1.12 (0.88, 1.43) | 0.345 |
| Q3 | 1.47 (1.17, 1.85) | 0.001 | 1.48 (1.18, 1.87) | 0.001 | 1.27 (1.00, 1.61) | 0.046 |
| Q4 | 1.93 (1.55, 2.40) | <0.001 | 1.94 (1.56, 2.42) | <0.001 | 1.41 (1.11, 1.78) | 0.004 |
| PHR for trend | 1.90 (1.55, 2.32) | <0.001 | 1.91 (1.56, 2.34) | <0.001 | 1.39 (1.13, 1.72) | 0.002 |

Model 1: not adjusted.

Model 2: adjusted for age and gender.

Model 3: adjusted for age, gender, residence, education, smoking, alcohol consumption, BMI, TG, LDL-C, Scr, hypertension, and heart disease.

PHR, platelet-to-high-density lipoprotein cholesterol ratio; HR, hazard ratio; CI, confidence interval;

BMI, body mass index; TG, triglycerides; Scr, serum creatinine; LDL-C, low-density lipoprotein cholesterol.
