# Supplementary material for: Association between platelet-to-high-density lipoprotein cholesterol ratio and incident diabetes risk: a national representative cohort study based on CHARLS
Source: BMC Endocr Disord. 2026 Oct 12;26:347. doi: 10.1186/s12902-026-02627-x (PMC13733031; doi:10.1186/s12902-026-02627-x)
Supplement: Supplementary file 5 [file 12902_2026_2627_MOESM5_ESM.docx]

**Table S5. Association Between PHR and Incident Diabetes After Excluding Extreme Platelet or HDL-C Values**

Extreme values were defined as platelet count or HDL-C values below the 10th percentile or above the 90th percentile.

| Exposure | Model 1 |  | Model 2 |  | Model 3 |  |
| --- | --- | --- | --- | --- | --- | --- |
|  | HR(95% CI) | P | HR(95% CI) | P | HR(95% CI) | P |
| PHR | 1.13 (1.09, 1.17) | <0.001 | 1.13 (1.09, 1.17) | <0.001 | 1.06 (1.02, 1.11) | 0.001 |
| PHR quartile |  |  |  |  |  |  |
| Q1 | 1.0 |  | 1.0 |  | 1.0 |  |
| Q2 | 1.18 (0.93, 1.50) | 0.137 | 1.18 (0.93, 1.51) | 0.174 | 1.10 (0.86, 1.40) | 0.437 |
| Q3 | 1.45 (1.15, 1.82) | 0.001 | 1.46 (1.15, 1.84) | 0.002 | 1.24 (0.98, 1.58) | 0.070 |
| Q4 | 1.86 (1.49, 2.33) | <0.001 | 1.88 (1.50, 2.35) | <0.001 | 1.38 (1.09, 1.75) | 0.008 |
| PHR for trend | 1.16 (1.10, 1.21) | <0.001 | 1.16 (1.10, 1.21) | <0.001 | 1.08 (1.02, 1.13) | 0.005 |

Model 1: not adjusted.

Model 2: adjusted for age and gender.

Model 3: adjusted for age, gender, residence, education, smoking, alcohol consumption, BMI, TG, LDL-C, Scr, hypertension, and heart disease.

PHR, platelet-to-high-density lipoprotein cholesterol ratio; HR, hazard ratio; CI, confidence interval;

BMI, body mass index; TG, triglycerides; Scr, serum creatinine; LDL-C, low-density lipoprotein cholesterol; HDL-C, high-density lipoprotein cholesterol.
