# Supplementary material for: Association between platelet-to-high-density lipoprotein cholesterol ratio and incident diabetes risk: a national representative cohort study based on CHARLS
Source: BMC Endocr Disord. 2026 Oct 12;26:347. doi: 10.1186/s12902-026-02627-x (PMC13733031; doi:10.1186/s12902-026-02627-x)
Supplement: Supplementary file 6 [file 12902_2026_2627_MOESM6_ESM.docx]

**Table S6. Baseline Characteristics of Included Participants and Otherwise Eligible Participants Excluded Due to Missing Data**

| Characteristic | Included participants N = 5835 | Excluded participants due to missing data N = 3768 |
| --- | --- | --- |
| Age, years, M(Q1,Q3) | 57 (51, 63) | 56 (50, 62) |
| Gender, n(%) |  |  |
| Male | 2624 (44.97%) | 1922 (51.01%) |
| Female | 3211 (55.03%) | 1846 (48.99%) |
| Residence, n(%) |  |  |
| Urban | 769 (13.18%) | 754 (20.01%) |
| Rural | 5066 (86.82%) | 3014 (79.99%) |
| Education, n(%) |  |  |
| Less than high school | 5282 (90.52%) | 3278 (87.0%) |
| High school and above | 553 (9.48%) | 490 (13.0%) |
| Smoke, n (%) |  |  |
| Never | 3638 (62.35%) | 2298 (60.99%) |
| Former | 445 (7.63%) | 264 (7.01%) |
| Current | 1752 (30.02%) | 1206 (32%) |
| Alcohol consumption, n(%) |  |  |
| Never | 4041 (69.25%) | 2562 (67.99%) |
| Former | 325 (5.57%) | 188 (5.0%) |
| Current | 1469 (25.18%) | 1018 (27.01%) |
| Hypertension, n(%) |  |  |
| No | 4559 (78.13%) | 3052 (81.0%) |
| Yes | 1276 (21.87%) | 716 (19.0%) |
| Heart disease, n (%) |  |  |
| No | 5242 (89.84%) | 3429 (91.0%) |
| Yes | 593 (10.16%) | 339 (9.0%) |
| BMI, kg/m2, M(Q1,Q3) | 23.0 (20.8, 25.6) | 23.0 (20.8, 25.5) |
| LDL-C, mg/dL, M(Q1,Q3) | 115 (94, 137) | 114 (92, 137) |
| TG, mg/dL, M(Q1,Q3) | 103 (74, 149) | 104 (74, 154) |
| Scr, mg/dL, M(Q1,Q3) | 0.75 (0.64, 0.87) | 0.77 (0.66, 0.88) |

Excluded participants were otherwise eligible participants without baseline diabetes who were excluded from the primary analysis because of missing data.

M, median; BMI, body mass index; TG, triglycerides; Scr, serum creatinine; LDL-C, low-density lipoprotein cholesterol.
