# Supplementary material for: Association between platelet-to-high-density lipoprotein cholesterol ratio and incident diabetes risk: a national representative cohort study based on CHARLS
Source: BMC Endocr Disord. 2026 Oct 12;26:347. doi: 10.1186/s12902-026-02627-x (PMC13733031; doi:10.1186/s12902-026-02627-x)
Supplement: Supplementary file 7 [file 12902_2026_2627_MOESM7_ESM.docx]

**Table S7. Association Between PHR and Incident Diabetes Using Multiple Imputation**

| Exposure | Model 1 |  | Model 2 |  | Model 3 |  |
| --- | --- | --- | --- | --- | --- | --- |
|  | HR(95% CI) | P | HR(95% CI) | P | HR(95% CI) | P |
| PHR | 1.10 (1.08, 1.13) | <0.001 | 1.10 (1.08, 1.13) | <0.001 | 1.06 (1.03, 1.09) | <0.001 |
| PHR quartile |  |  |  |  |  |  |
| Q1 | 1.0 |  | 1.0 |  | 1.0 |  |
| Q2 | 1.12 (0.82, 1.51) | 0.455 | 1.12 (0.82, 1.52) | 0.441 | 1.08 (0.79, 1.47) | 0.593 |
| Q3 | 1.32 (1.07, 1.64) | 0.011 | 1.33 (1.07, 1.65) | 0.010 | 1.22 (0.98, 1.53) | 0.072 |
| Q4 | 1.81 (1.48, 2.22) | <0.001 | 1.82 (1.49, 2.23) | <0.001 | 1.48 (1.18, 1.86) | 0.001 |
| PHR for trend | 1.15 (1.11, 1.19) | <0.001 | 1.15 (1.11, 1.20) | <0.001 | 1.10 (1.05, 1.14) | <0.001 |

Missing covariate data were imputed using multiple imputation with five imputed datasets. Cox proportional hazards regression models were fitted separately in each imputed dataset, and estimates were pooled according to Rubin’s rules.

Model 1: not adjusted.

Model 2: adjusted for age and gender.

Model 3: adjusted for age, gender, residence, education, smoking, alcohol consumption, BMI, TG, LDL-C, Scr, hypertension, and heart disease.

PHR, platelet to high density lipoprotein cholesterol ratio; HR, hazard ratio; CI, confidence interval;

BMI, body mass index; TG, triglycerides; Scr, serum creatinine; LDL-C, low-density lipoprotein cholesterol.
